# Identification of Key Candidate Genes in Dairy Cow in Response to *Escherichia coli* Mastitis by Bioinformatical Analysis

**DOI:** 10.3389/fgene.2019.01251

**Published:** 2019-12-06

**Authors:** Liabin Li, Xiuli Chen, Zeshi Chen

**Affiliations:** ^1^Key Laboratory of Tropical Animal Breeding and Epidemic Disease Research of Hainan Province, College of Animal Science and Technology, Hainan University, Haikou, China; ^2^Animal Disease Prevention and Control Center of Hanzhong, Hanzhong, China

**Keywords:** bovine mastitis, *Escherichia coli*, differentially expressed gene, pathway, biomarker

## Abstract

At present, bovine mastitis is one of the most costly diseases affecting animal health and welfare. *Escherichia coli* (*E. coli*) is considered to be one of the main pathogens causing mastitis with clinical signs in dairy cattle. However, the cure rate of *E. coli* mastitis is low, and the pathogenesis of *E. coli* mastitis is not completely known. In order to develop new strategies for the rapid detection of *E. coli* mastitis, a comprehensive molecular investigation of *E. coli* mastitis is necessary. Hence, this study integrated three microarray data sets to identify the potential key candidate genes in dairy cow in response to *E. coli* mastitis. Differentially expressed genes (DEGs) were screened in mammary gland tissues with live *E. coli* infection. Furthermore, the pathways enrichment of DEGs were analyzed, and the protein–protein interaction (PPI) network was performed. In total, 105 shared DEGs were identified from the three data sets. The DEGs were significantly enriched in biological processes mainly involved in immunity. The PPI network of DEGs was constructed with 102 nodes and 546 edges. The module with the highest score through MCODE analysis was filtered from PPI; 18 central node genes were identified. However, in addition to immune-related pathways, some of the 18 DEGs were involved in signaling pathways triggered by other diseases. Considering the specificity of biomarkers for rapid detection, *IL8RB, CXCL6*, and *MMP9* were identified as the most potential biomarker for *E. coli* mastitis. In conclusion, the novel DEGs and pathways identified in this study can help to improve the diagnosis and treatment strategies for *E. coli* mastitis in cattle.

## Introduction

Domesticated cattle not only provide a significant source of nutrition but also provide a livelihood for nearly 6.6 billion humans (Elsik et al., 2009). Bacterial infection is one of the most important enemies of the cattle farming industry, causing significant losses. For example, bovine mastitis caused mostly by bacterial infection, resulting in losses of up to $2 billion per year in the United States alone. Aside from the economic losses, mastitis can impair animal welfare, and poses a threat to human health since it may be responsible for transfer of antimicrobial resistance and for food poisoning (Johler et al., 2015; Käppeli et al., 2019). *Escherichia coli* (*E. coli*) which as a prevalent environmental pathogen that routinely colonizes dairy cattle is one of the main pathogens causing mastitis (Buitenhuis et al., 2011). *E. coli* often leads to severe clinical mastitis and induces a distinct acute phase response (APR) (Petzl et al., 2018).

To date, the conventional method of treatment for *E. coli* mastitis is the use of antibiotics. However, the treatment using antibiotics is often less than 50% effective and leads to premature culling in many cases (Schmelcher et al., 2015). Alternative options are urgently needed for *E. coli* mastitis treatment. A deeper understanding of the molecular basis of *E. coli* mastitis may uncover new ways in battling this costly disease. Moreover, the comprehensive molecular investigation of *E. coli* mastitis may aid in the identification of new biomarkers for the rapid detection, and personalized therapy.

Microarray is a gene detection technique; using microarray can quickly detect the gene expression information in animals under infectious disease, which is particularly suitable for differentially expressed gene (DEG) screening. With the widespread use of microarray technology, a large amount of raw data about gene expression has been generated, and most of the data have been stored in public databases. The integration and reanalysis of these raw data can provide valuable information for new researches (Guo et al., 2017). In recent years, many microarray data analysis studies on *E. coli* mastitis have been carried out, and hundreds of DEGs have been identified (Han, 2019). For instance, a study identified 928 DEGs involved in the mammary gland with *E. coli* mastitis (Buitenhuis et al., 2011). Another study found 2,154 DEGs in *E. coli* mastitis vs. control treatment (Mitterhuemer et al., 2010). The results of different independent studies are always limited by the samples with environment, breed, population, and specific animal differences. Therefore, it is difficult to use differential genes obtained in a single independent study as biomarkers of *E. coli* mastitis. So far, the performance of the most mastitis detection systems do not meet the high accuracy required for clinical diagnosis needs of mastitis in cattle (Jensen et al., 2016). The aim of developmental research is to enhance the diagnostic efficiency of bovine mastitis with including several biomarkers on one test strip. The integrated bioinformatics methods combining with microarray technique will be innovative and might promote the appearance of test strip containing several biomarkers.

There are many gene expression profiles of *E. coli*–treated samples in the NCBI–Gene Expression Omnibus (NCBI-GEO) database. Most of these studies use primary mammary epithelial cells as test subjects to obtain data, and a small part of them were *in vivo* experiments using cows. However, primary mammary epithelial cells experiments are of limited significance because the *in vitro* cell testing does not mimic the complex environment with interactions between pathogens, antimicrobials, and components of the host’s immune response inside the mammary gland. Therefore, we integrated microarray data sets obtained from *in vivo* experiments with live *E. coli* infection to identify key candidate genes and pathways in *E. coli* mastitis of cows. It is anticipated that these results may provide more accurate, practically reliable biomarkers for early diagnosis and individualized prevention and therapy of bovine *E. coli* mastitis.

## Materials and Methods

### Data Set Collection and Identification of DEGs

The transcription profile data sets of bovine mammary gland with or without live *E. coli* infection were downloaded from NCBI-GEO database (https://www.ncbi.nlm.nih.gov/gds/). The accession number was GSE15020, GSE24217, and GSE50685. These studies used the “Affymetrix Bovine Genome Array” platform GPL2112, which contains 24,128 genes. A total of 22 *E. coli* mastitis cases and 19 normal mammary gland data were obtained (**Table 1**). The raw data (.CEL files) of these three microarray data sets were downloaded.

**Table 1:** Summary of the microarray data sets included in the analysis.

Accession number	Treatment time (h)	Pathogen	Tissue	Samples* (Con: Tr)	Reference
GSE15020	24	*E. coli* 1303	Udder biopsy	5:5	(Mitterhuemer et al., 2010)
GSE24217	24	*E. coli* K2BH2	Udder biopsy	9:12	(Buitenhuis et al., 2011)
GSE50685	24 and 48	*E. coli* ECC-Z	Udder biopsy	5:5	(Sipka et al., 2014)

*The data of E. coli treatment (≥24 h) group and normal control group in each data set were selected; (Con: Tr), number of healthy samples: number of treatment samples.

R software (Version 3.5.1; https://www.r-project.org/), affy package (https://bioconductor.org/packages/release/bioc/html/affy.html), and affyPLM (http://bioconductor.org/packages/release/bioc/html/affyPLM.html) package were used for raw data analysis (Li et al., 2019). The raw data in CEL format were converted into expression measures. Log scale robust multi-array analysis (RMA) background correction, quantile normalization, pmonly (perfect match correction), and median polish were performed in the R software (Li et al., 2019). RMA functions are provided by affy package (Irizarry et al., 2003). After these processes, we got the gene expression matrix. Eventually, the Linear Models for Microarray Data (LIMMA; http://www.bioconductor.org/packages/release/bioc/html/limma.html) package from Bioconductor was applied to identify DEGs by comparing expression value in mammary gland with or without *E. coli* infection. DEGs were identified with classical t test. A |log_2_ fold change (FC)| > 1 and *P* value < 0.05 were regarded as the cutoff criterion for DEGs.

### Enrichment Analyses of DEGs

In this study, notably relevant Kyoto Encyclopedia of Genes and Genomes (KEGG) pathway and Gene Ontology (GO) analyses were carried out using the DAVID (Database for Annotation, Visualization and Integrated Discovery) Bioinformatics Resources 6.8 (https://david.ncifcrf.gov/). *P* < 0.01 was chosen as the cutoff criteria. R software was used for data visualization.

### Identification of Key Candidate Genes With Protein–Protein Interaction (PPI) Network Analysis and Module Mining

The STRING database (Version 11.0; https://string-db.org/) was used to annotate functional interactions between DEGs and other genes. Based on this information, PPI network was visualized by Cytoscape (Version 3.7.1) (Smoot et al., 2010). Then Cytoscape plugin: MCODE and CentiScape were used to search modules of highly inter-connected nodes from the PPI network complex. Moreover, the function and pathway enrichment analysis of DEGs in the modules were performed by using DAVID.

## Results

### Identification of DEGs

**Figure 1:**
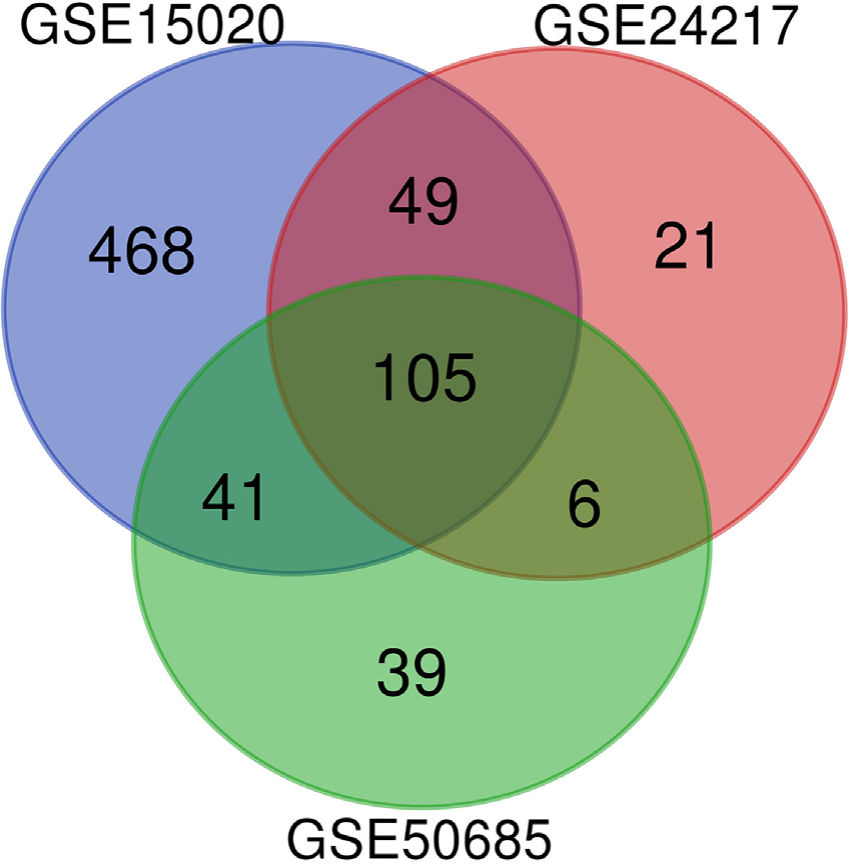
The DEGs between (*E. coli*) mastitis and normal tissues in three original microarray data sets (GSE15020, GSE24217, and GSE50685) were identified.

To identify DEGs in *E. coli* mastitis, three original microarray data sets were downloaded from NCBI-GEO database. Using the calculating criteria of *P* < 0.05 and absolute log_2_ FC > 1, we extracted 663, 181, and 192 DEGs from the expression profile data sets GSE15020, GSE24217, and GSE50685, respectively. Employing R software and ggplot2 package, we developed Volcano Plot of the DEGs for the three expression profile data sets (**Figure S1**). The genes identified are in the same direction from each study. Next, we integrated the three groups of DEGs and performed bioinformatics analysis. The results are shown in **Figure 1**. A total of 105 DGEs were identified from the three profile data sets, including 98 up-regulated genes, 6 down-regulated genes, and 1 aberrantly expressed gene *SLC2A3* in the *E. coli* treatment samples compared to healthy samples (**Table 2**).

**Table 2:** One hundred and five DEGs were identified from the three profile data sets, including 98 up-regulated genes, 6 down-regulated genes, and 1 aberrantly expressed gene SLC2A3 in the *E. coli* treatment samples, compared to healthy samples.

DEGs	Gene Name
Up-regulated	*VCAM1, VASP, UGDH, TXN, TUBB, TREM1, TNFRSF1A, TNFAIP6, TLR2, TIMP1, SOCS3, SLC1A1, SLC11A1, SERPINE2, SERPINE1, SERPINA3, SELL, SCIN, SAA3, S100A4, S100A12, RETN, RAC2, QKI, EIF2AK2, PRDX5, PLAUR, PLAU, PLA2G7, PCTP, NTS, NT5C2, NFKBIA, NCF2, NCF1, MX1, MT1A, MMP9, LOC407171, LGALS1, LAP, ITGB6, ITGB2, ITGA2, ISG15, IL8RB, CXCL8, IL6, IL2RA, IL1RN, IL1B, IL1A, IL18, IFNAR2, IFITM3, ICAM3, ICAM1, GNAI2, GNAI1, GAPDH, F5, ETS2, DNASE2, DGAT2, DEFB1, CYBA, CXCL6, CXCL2, GRO1, CTSZ, CTSS, CTSC, VCAN, CORO1A, CHI3L1, CD97, CD69, CD44, CCL3, CCL20, CCL2, CASP4, CAPG, CA2, BPIFB1, TSPO, DEFB5, PYCARD, ARRB1, ARG2, ARF2, ANXA1, ADAMTS4, ADA, ACADVL, BNBD-9-LIKE, BASP1, GPX1*
Down-regulated	*SPADH1, SLC38A3, MUC15, CNGA1, ACSM1, ALOX15*

### Functional and Pathway Enrichment Analysis

**Figure 2:**
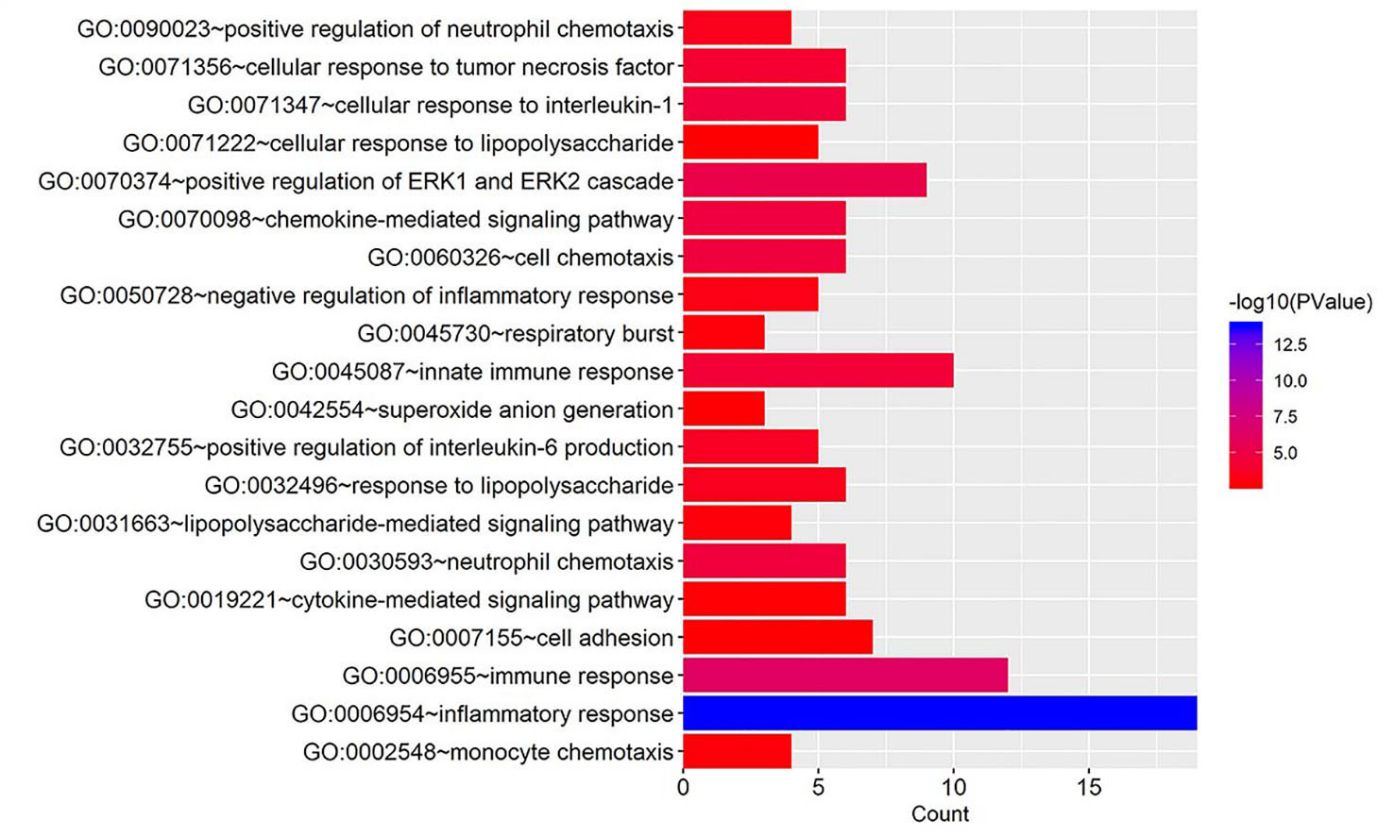
Gene ontology analysis of DEGs associated with *E. coli* mastitis. The biological process (BP) in functional enrichment of DEGs was performed using the online biological tool, DAVID, with count and *P* value.

**Figure 3:**
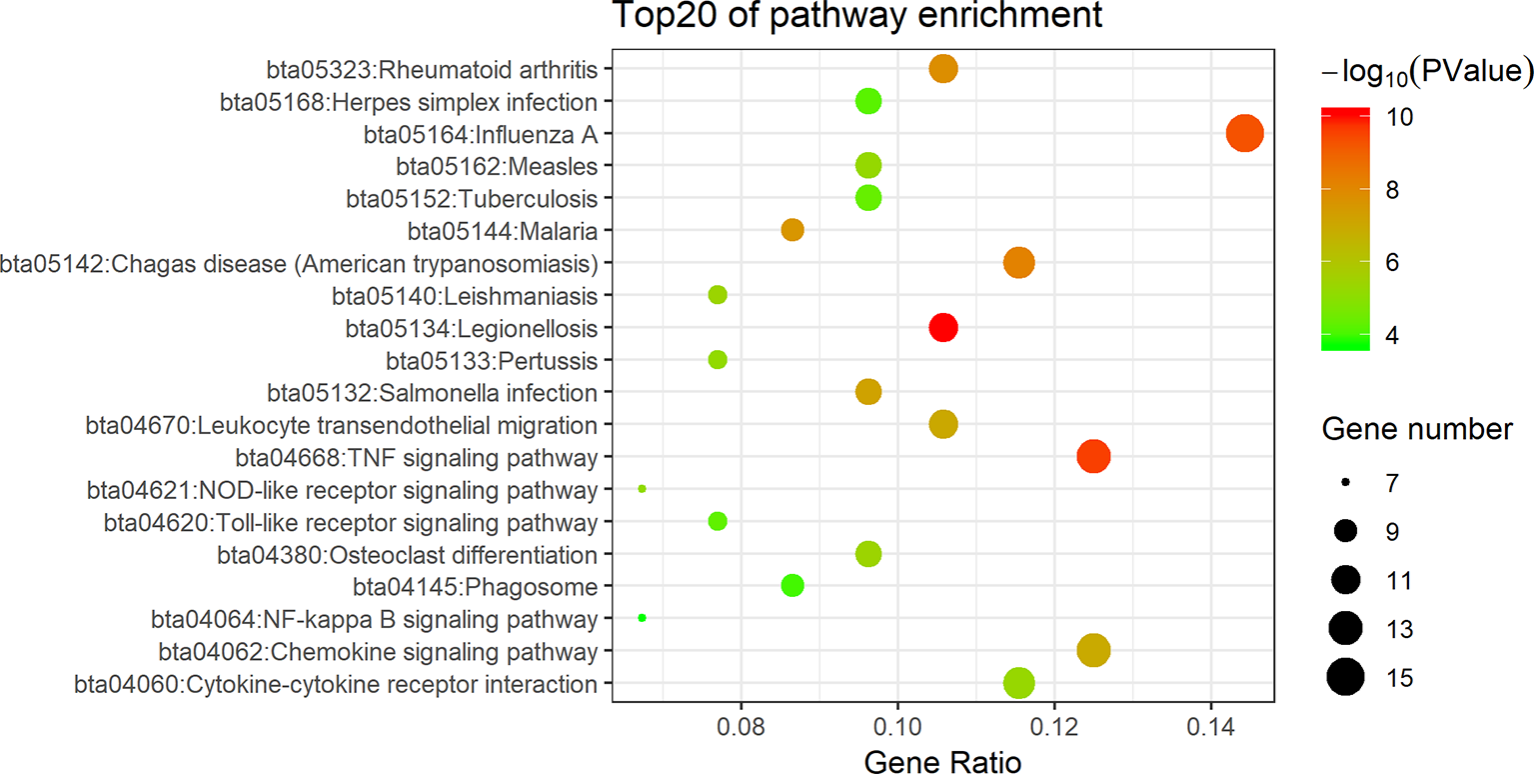
Signaling pathway analysis of DEGs associated with *E. coli* mastitis. The Kyoto Encyclopedia of Genes and Genomes (KEGG) pathways enrichment of DEGs was performed using the online biological tool DAVID with gene ratio, gene number, and *P* value.

To acquire further understanding of the functions of identified DEGs, all DEGs were uploaded to DAVID. GO biological process (BP) terms and KEGG pathways were enriched for 105 candidate DEGs in *E. coli* mastitis. The top 20 BP terms according to *P* value are shown in **Figure 2**. The data showed that DEGs were mainly involved in GO terms about immunity such as inflammatory response, immune response, innate immune response. DEGs mainly enriched in extracellular space, extracellular exosome, extracellular region, and external side of plasma membrane in the molecular function term. In these candidate DEGs of *E. coli* mastitis, 27 KEGG pathways were found to be enriched with *P* < 0.05 as the cutoff point, such as the TNF signaling pathway, chemokine signaling pathway, and cytokine–cytokine receptor interaction (**Figure 3**).

### Identification of Key Candidate Genes With PPI Network Analysis and Modular Analysis

**Figure 4:**
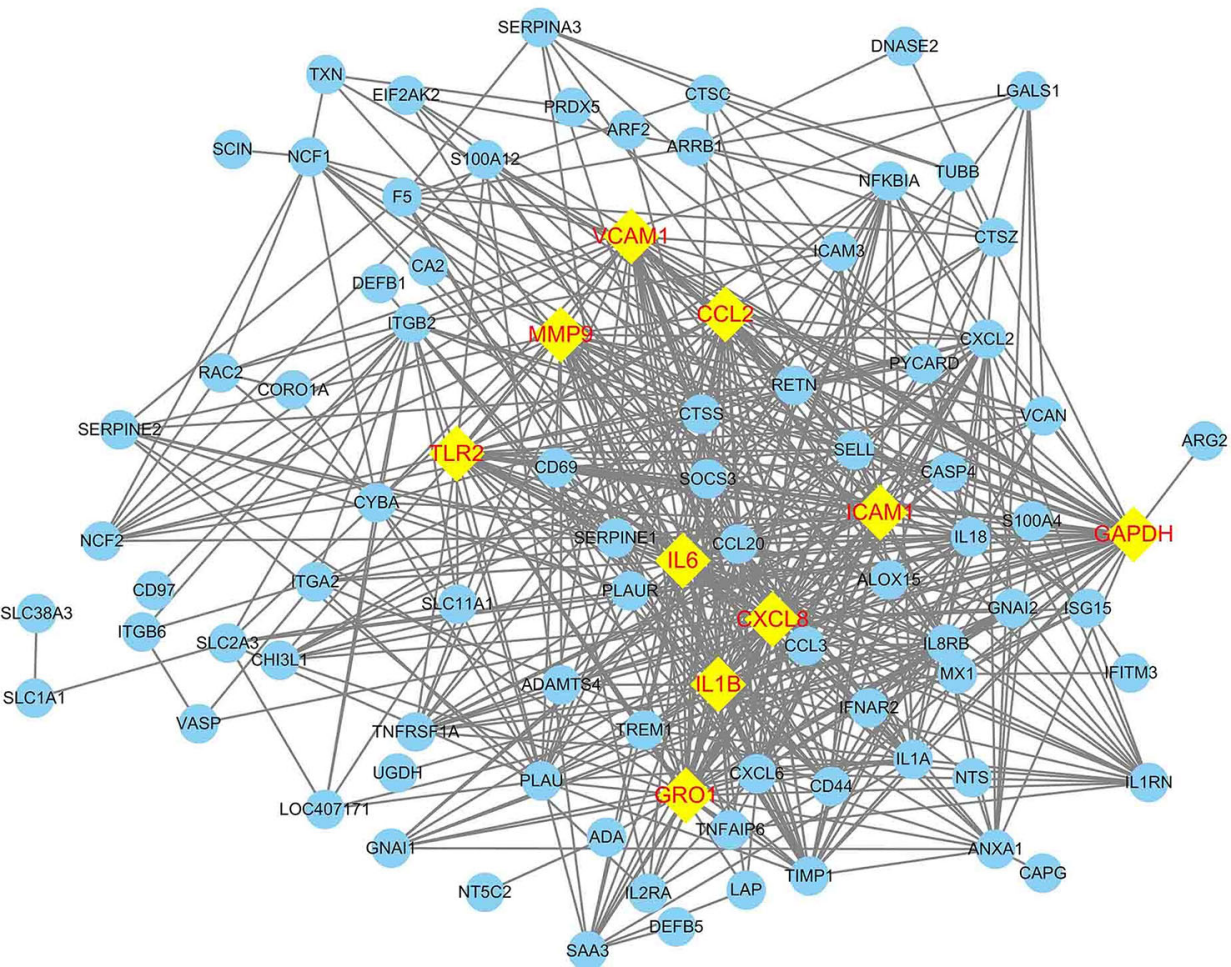
Construction of protein–protein interaction (PPI) network of DEGs associated with *E. coli* mastitis. The nodes with higher degrees were shaped as diamond in yellow.

**Figure 5:**
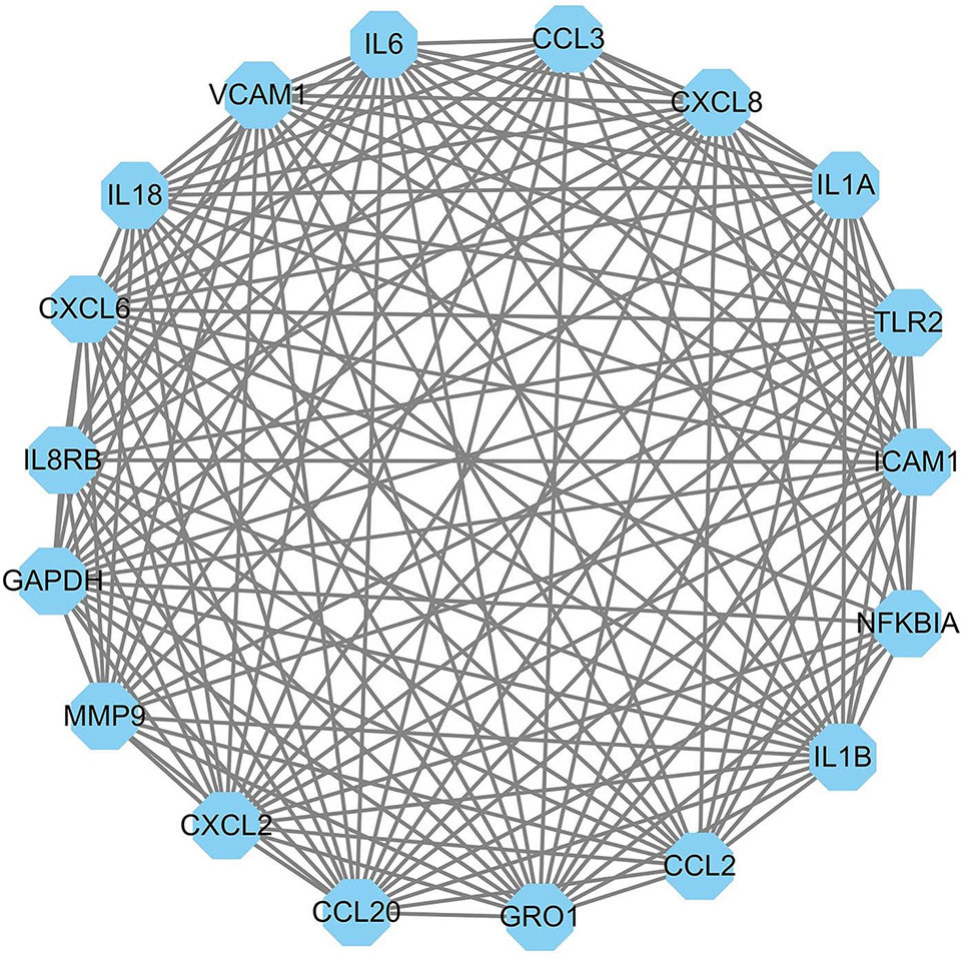
The most significant module of the PPI network complex of DEGs associated with *E. coli* mastitis. The module consists of 18 nodes and 144 edges, which are mainly associated with immune response.

The PPI network of DEGs was constructed in the STRING database which has 102 nodes and 546 edges. A total of 102 DEGs (except *BASP1, BNBD-9-LIKE*, and *GPX1*) were filtered into the DEGs PPI network complex. The PPI network was visualized by using Cytoscape software (**Figure 4**). Based on the STRING database, the DEGs with the highest PPI scores identified by the Cytoscape plugin: MCODE and CentiScape with three centrality methods were shown in **Table 3**. Among the 102 nodes, 37 central node genes were identified with the filtering of node degree >10. The most significant 10 node degree genes were shaped as diamond with yellow in **Figure 4**. The module with the highest score through MCODE analysis was filtered from PPI. This module consisted of 18 nodes and 144 edges (**Figure 5**), which are mainly associated with inflammatory response, immune response, TNF signaling pathway, and rheumatoid arthritis, etc. (**Tables 4** and **5**). All the 18 genes belong to high PPI score genes (**Table 3**). The log_2_ FC of these genes in three microarray data sets were summarized, and the top 10 genes with high log_2_ FC were listed in **Table 6**. These genes are significantly up-regulated in *E. coli* mastitis tissues. These genes are key genes associated with *E. coli* mastitis.

**Table 3:** The DEGs with PPI scores >10 identified by the MCODE and CentiScape with three centrality methods.

Gene name	Score	Degree	Betweenness	Closeness
***MMP9***	13.76666667	36	507.3346801	0.006666667
***IL18***	13.76666667	24	55.68803225	0.005988024
***GAPDH***	13.58823529	48	1866.518572	0.007575758
***CXCL8***	13.58823529	44	587.0335144	0.007092199
***IL6***	13.58823529	44	689.8891114	0.007142857
***IL1B***	13.58823529	37	614.3307106	0.006666667
***TLR2***	13.58823529	36	439.7926335	0.006756757
***GRO1***	13.58823529	33	279.4289156	0.006535948
***ICAM1***	13.58823529	29	182.6316596	0.006369427
***VCAM1***	13.58823529	29	191.3593862	0.006451613
***CXCL2***	13.58823529	27	185.2823819	0.00625
***CCL20***	13.58823529	22	50.82378679	0.00591716
***CXCL6***	13.58823529	22	35.29524062	0.00591716
***IL8RB***	12.87619048	23	139.4002054	0.005988024
***IL1A***	12.87619048	21	30.6506403	0.00591716
***CCL3***	12.675	19	11.09228542	0.005780347
***CCL2***	12.06535948	33	142.3940048	0.006451613
***NFKBIA***	12	16	16.59909809	0.005617978
***IL1RN***	10.51648352	18	26.25812678	0.005524862
***TIMP1***	10.26666667	25	170.4422329	0.006134969

**Table 4:** Gene ontology analysis BP of genes in selected module.

Term	Description	P-value	Genes
**GO:0006954**	Inflammatory response	6.48E−11	*CCL3, CCL2, CCL20, IL18, CXCL2, TLR2, CXCL8, GRO1*
**GO:0006955**	Immune response	6.48E−11	*CCL3, CCL2, IL18, CXCL2, IL1B, CXCL8, GRO1, IL1A*
**GO:0070098**	Chemokine-mediated signaling pathway	1.55E−09	*CCL3, CCL2, CCL20, CXCL2, CXCL8*
**GO:0071347**	Cellular response to interleukin-1	2.70E−07	*CCL3, IL6, CCL2, CCL20, CXCL8*
**GO:0060326**	Cell chemotaxis	2.70E−07	*CCL2, CCL20, CXCL2, GRO1*
**GO:0071356**	Cellular response to tumor necrosis factor	6.37E−07	*CCL3, IL6, CCL2, CCL20, CXCL8*
**GO:0031663**	Lipopolysaccharide-mediated signaling pathway	4.97E−06	*CCL3, CCL2, IL18, NFKBIA*
**GO:0071346**	Cellular response to interferon-gamma	1.08E−05	*CCL3, CCL2, CCL20, GAPDH*
**GO:0030593**	Neutrophil chemotaxis	2.42E−05	*CCL3, CCL2, CCL20, CXCL8*
**GO:0032496**	Response to lipopolysaccharide	1.34E−04	*CXCL2, NFKBIA, CXCL8*

**Table 5:** KEGG pathway analysis of genes in selected module.

Term	Description	P-Value	Genes
**bta04668**	TNF signaling pathway	4.17E−15	*VCAM1, ICAM1, IL6, CCL2, CCL20, MMP9, CXCL2, IL1B, NFKBIA, GRO1*
**bta05323**	Rheumatoid arthritis	1.20E−13	*ICAM1, CCL3, IL6, CCL2, CCL20, IL18, TLR2, IL1B, CXCL8, IL1A*
**bta05134**	Legionellosis	1.46E−13	*IL6, IL18, CXCL2, TLR2, IL1B, NFKBIA, CXCL8, GRO1*
**bta05132**	Salmonella infection	3.39E−12	*CCL3, IL6, IL18, CXCL2, IL1B, CXCL8, GRO1, IL1A*
**bta05144**	Malaria	1.03E−11	*VCAM1, ICAM1, IL6, CCL2, IL18, TLR2, IL1B, CXCL8*
**bta05164**	Influenza A	1.32E−09	*ICAM1, IL6, CCL2, IL18, IL1B, NFKBIA, CXCL8, IL1A*
**bta05142**	Chagas disease	2.55E−09	*CCL3, IL6, CCL2, TLR2, IL1B, NFKBIA, CXCL8*
**bta04060**	Cytokine–cytokine receptor interaction	8.26E−09	*CCL3, IL6, CCL2, CCL20, IL18, IL1B, CXCL8, IL1A*
**bta04621**	NOD-like receptor signaling pathway	6.70E−08	*IL6, CCL2, IL18, IL1B, NFKBIA, CXCL8*
**bta04620**	Toll-like receptor signaling pathway	6.85E−08	*CCL3, IL6, TLR2, IL1B, NFKBIA, CXCL8*
**bta04062**	Chemokine signaling pathway	7.49E−08	*CCL3, CCL2, CCL20, CXCL2, NFKBIA, CXCL8, GRO1*

**Table 6:** The top 10 genes in selected module with high log_2_ FC.

Genes	Log_2_ FC			
	**GSE50685**	**GSE24217**	**GSE15020**	**Mean ± SD**
***CXCL8***	5.1324262	4.8830127	8.2002277	6.07 ± 1.85
***CXCL2***	4.5813085	5.3235659	7.2746447	5.73 ± 1.39
***IL8RB***	4.4244941	5.9182538	6.733066	5.69 ± 1.17
***CXCL6***	4.2105549	4.3959378	5.7311918	4.78 ± 0.83
***GRO1***	3.0088492	3.5571272	4.8596901	3.81 ± 0.95
***IL6***	2.5340753	2.9049886	3.8233124	3.09 ± 0.66
***IL1B***	1.3651293	2.8089291	3.2705019	2.48 ± 0.99
***MMP9***	1.1651751	2.6356085	3.5852836	2.46 ± 1.22
***ICAM1***	2.199757	2.1063726	2.9454883	2.42 ± 0.46
***TLR2***	1.7205774	1.7875642	3.0374119	2.18 ± 0.74

## Discussion

Despite the research on mastitis having lasted for a long time, the detrimental economic losses caused by mastitis remains unchanged (Petzl et al., 2018). Early diagnosis is very important due to the high costs of mastitis. However, most current detection systems do not meet the high accuracy required for clinical diagnosis needs of mastitis (Jensen et al., 2016). Nowadays, somatic cell count (SCC) and California mastitis test (CMT) are often used in the diagnosis of mastitis. Mastitis can cause significant milk changes such as the presence of clots in milk, milk discoloration, and high levels of leukocyte numbers which lead to a rise in SCC. Therefore, SCC has been used as the gold standard for decades to diagnose subclinical mastitis. As SCC requires submission of sample to a laboratory for automated cell counting, it’s very time-consuming. In addition, SCCs do not always correlate with mastitis, and they may be affected by other factors (e.g., lactation number, stage of lactation, milk production level, stress, season, and breed) (Duarte et al., 2015). CMT is based on the principle of the addition of a detergent to a milk sample with a high cell count which promotes cell lysis, nucleic acid release, and formation of a “gel-like” matrix. The CMT is quick, cheap, and simple, but the interpretation can be subjective, and this might result in false positives and negatives (Viguier et al., 2009).

Therefore, it is urgent to develop a new diagnosis system which could be adapted to rapid, on-farm diagnostic systems. Development of new biomarkers for diagnosis of mastitis has been considered as the trend of mastitis detection (Duarte et al., 2015). A biomarker is a characteristic that can be measured and evaluated as an indicator of pathological processes, or pharmacological responses to therapeutic interventions. Genes coding for proteins such as haptoglobin, cathelicidin antimicrobial peptide, and lingual antimicrobial peptide have been identified as potential biomarkers for mastitis detection (Sharifi et al., 2018). At present, antibiotics are mainly used as systemic treatment of mastitis (Porter et al., 2016). Antibiotic should be chosen based on specific mastitis pathogen. Therefore, the exploration of biomarkers for a specific pathogen is also conducive to the selection of appropriate antibiotic therapy. Combined with previous studies on biomarkers, the genes detected in this experiment have the potential to develop test strips which include several biomarkers on one test strip, and this can enhance the diagnostic efficiency.

Here, we performed bioinformatical analysis on three microarray profile data sets to identify key genes that may be significantly involved in response to bovine *E. coli* mastitis. To obtain unique genes and pathways associated with *E. coli* mastitis, for the first time, we only selected original microarray data sets obtained from *in vivo* experiments with live *E. coli* infection. The results confirmed the most important findings in previous individual studies such as induction of the responses related to immune response, inflammation, and TNF signaling pathway (Buitenhuis et al., 2011; Günther et al., 2011). In the current research, the top-ranked genes were *CXCL8*, *CXCL2*, *IL8RB*, *CXCL6*, *GRO1*, *IL6*, *IL1B*, *MMP9*, *ICAM1*, and *TLR2* (**Table 6**). This result similar to previous studies such as Sharifi et al. (2018), who found *CXCL8*, *CXCL2*, and *GRO1* were the top three genes associated with *E. coli* mastitis, and Han (2019) found *CXCL2* and *GRO1* were key genes in both the *E. coli* and the *Staphylococcus aureus* mastitis. The consistency of these key genes proves the validity of our study.

Importantly, new key genes and pathways associated with *E. coli* mastitis were obtained by this bioinformatical analysis. *IL8RB* and *CXCL6* are key genes newly identified through our analysis. More importantly, for the first time, this study found that the DEGs associated with *E. coli* mastitis were involved in signaling pathways triggered by other diseases (**Table 5**). For example, *CXCL8*, *CXCL2*, or *GRO1* were involved in rheumatoid arthritis, legionellosis, salmonella infection, and so on. The new finding may contribute to understanding the molecular basis of mastitis pathogenesis. However, this also indicates that those genes may not be suitable as a molecular biomarker of *E. coli* mastitis on account of biomarker should be specific for a disease and should remain unchanged by unrelated disorders. Therefore, after removing genes that cross with other diseases, only *IL8RB*, *CXCL6*, and *MMP9* have the potential to be biomarker of *E. coli* mastitis in this study.

Interleukin 8 (*IL8*) is an important chemokine and plays a major role in the recruitment of neutrophils and lymphocytes from peripheral sites to the mammary gland during *E. coli* mastitis. *IL8* has been confirmed to be produced by epithelial cells in the mammary gland respond to *E. coli* infection (Boehmer et al., 2008). However, these effects of *IL8* must be achieved by binding to the corresponding receptors; during this process, *IL8RB* is the receptor gene of *IL8*. Moreover, *IL8RB* has exhibited an important role in immune function during mastitis infection, and it belongs to the promising candidate genes contributing in bovine mastitis (El Nahas et al., 2017). Consistent with the above research, in this study, *IL8RB* was identified as a key gene in response to *E. coli* mastitis. Therefore, its role during *E. coli* mastitis should be further studied.

Chemokines are a family of proteins with diverse functions that mediate a variety of inflammatory reactions (Gray et al., 2005). Chemokine ligand 6 (*CXCL6*) is mainly secreted by macrophages, epithelial cells, and stromal cells and has chemotactic granulocytes and antimicrobial and immune functions. *CXCL6* is also known as granulocyte chemotactic protein 2 (GCP-2). There was evidence that indicated the up-regulation of *CXCL6* gene expression in bovine mammary epithelial cell line with bacterial infection (Yu et al., 2010) and in infected gland (Rinaldi et al., 2010). Combined with the analysis of this study, it shows again that *CXCL6* plays an important role in cow responses to *E. coli* mastitis.

Matrix metalloproteinases (MMPs) are a family of calcium-dependent zinc-containing endopeptidases which are synthesized and secreted by various cells, such as neutrophils and macrophages, and play important roles in inflammation (Vandooren et al., 2013) such as bovine mammary epithelial cell with *E. coli* mastitis (Zhao and Lacasse 2008). This family of proteases have five subfamilies, of which *MMP9* is one of the most studied. It has been shown that the levels of expression of *MMP9* in cases of bovine *E. coli* mastitis (Caggiano et al., 2019) and in goat mammary gland epithelial cells with *S. aureus* infection (Li et al., 2016) were increased. Considering all the results, *MMP9* may be a good candidate for biomarker of *E. coli* mastitis diagnosis.

In view of the fact that the performances of the most current bovine mastitis detection systems do not meet the high accuracy required for clinical diagnosis needs of mastitis (Jensen et al., 2016), the main purpose of this study is to identify potential genes that can be used as biomarkers for the diagnosis of *E. coli* mastitis. Although these genes have been identified in this study and had been preliminarily confirmed in previous animal or cell experiments, these genes still need more laboratory-based studies and farm practice to confirm.

## Conclusions

A total of 105 DEGs related to mastitis were identified from microarray data sets of *in vivo* experiments with live *E. coli* infection. After further integrated bioinformatical analysis, *IL8RB*, *CXCL6*, and *MMP9* were identified as the most potential biomarker for *E. coli* mastitis. In conclusion, this study provided an extensive bioinformatics analysis of DEGs and revealed a series of targets and pathway for further study to battle with *E. coli* mastitis.

## Data Availability Statement

Publicly available datasets were analyzed in this study. This data can be found here: GSE15020, GSE24217, GSE50685.

## Author Contributions

LL designed the experiments. LL, XC, and ZC did the data analysis and wrote the paper. All authors read and approved the final manuscript.

## Funding

This work was supported by a Startup Fund of Hainan University [KYQD(ZR) 1940] to LL.

## Conflict of Interest

The authors declare that the research was conducted in the absence of any commercial or financial relationships that could be construed as a potential conflict of interest.
