# Supplementary material for: Identification of Key Candidate Genes in Dairy Cow in Response to Escherichia coli Mastitis by Bioinformatical Analysis
Source: Front Genet. 2019 Dec 6;10:1251. doi: 10.3389/fgene.2019.01251 (PMC6915111; doi:10.3389/fgene.2019.01251)
Supplement: Supplementary file 1 [file Presentation_1.pdf]

Supplementary Material

Supplementary Figure 1

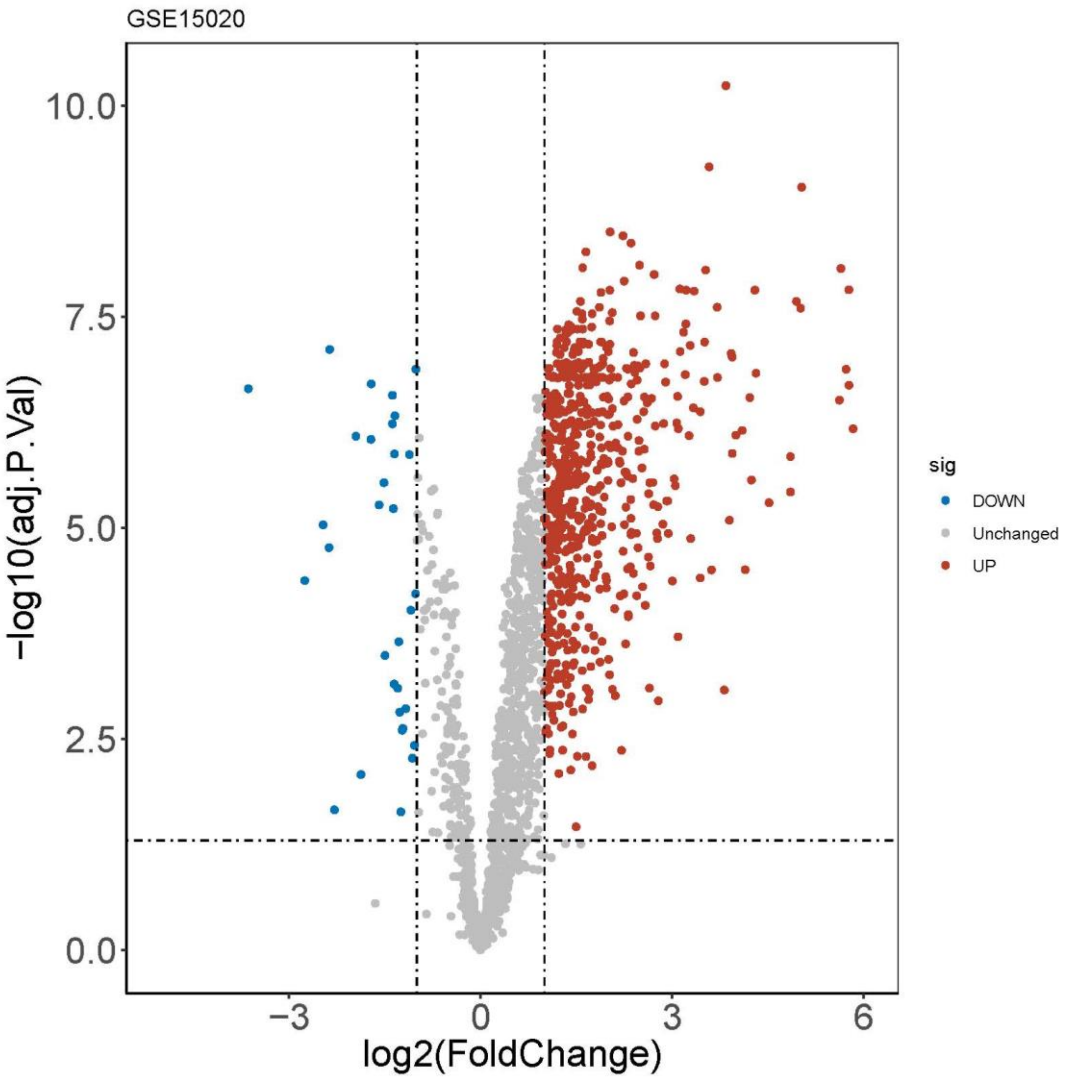

Supplementary Figure 1A. Volcano plot of the differentially expressed genes (DEGs) in GSE15020. The red and blue dots indicated significantly upregulated or downregulated expression of genes in mammary gland tissues with live *E. coli* infection compared to normal tissues.

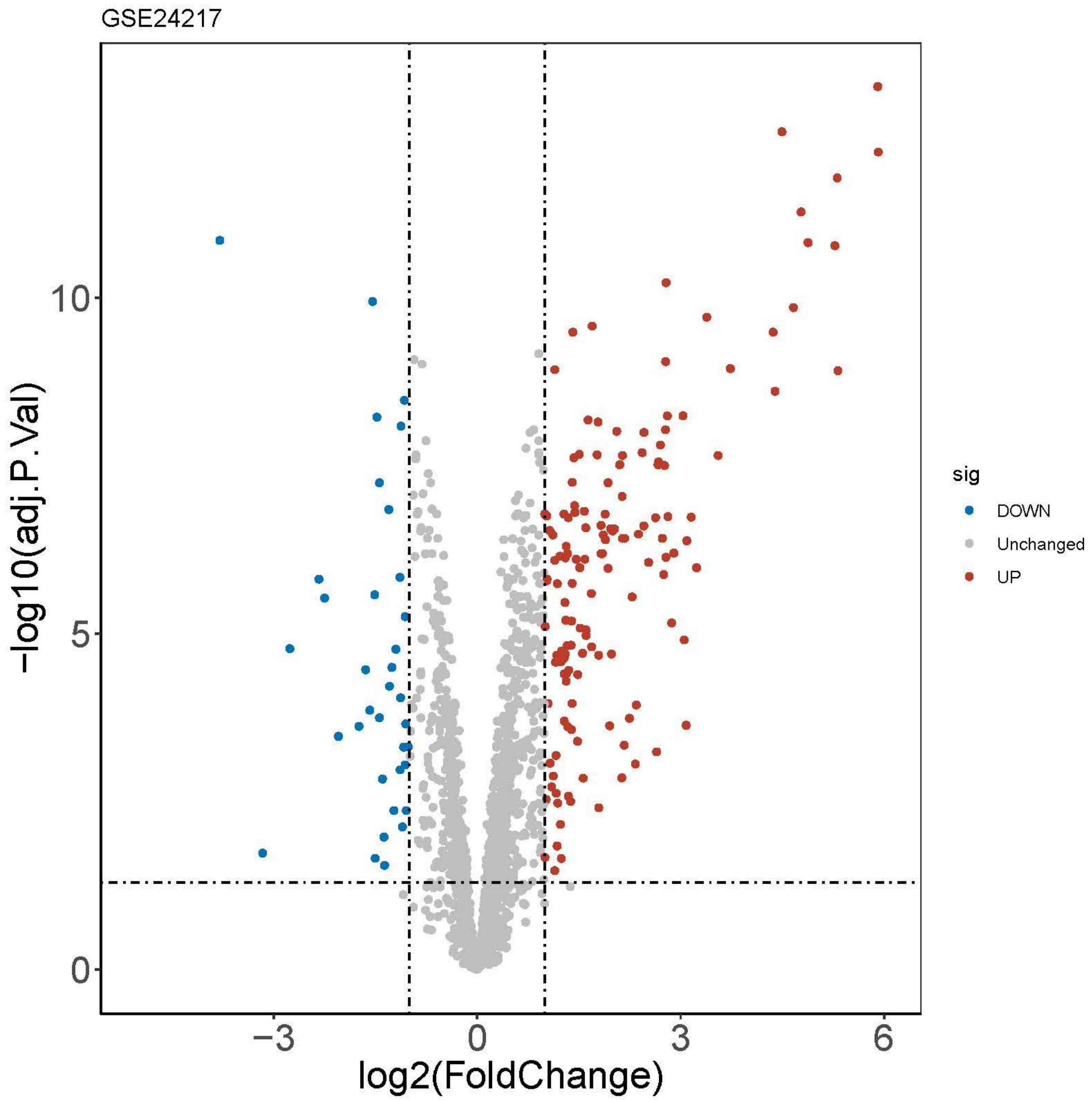

Supplementary Figure 1B. Volcano plot of the DEGs in GSE24217. The red and blue dots indicated significantly upregulated or downregulated expression of genes in mammary gland tissues with live *E. coli* infection compared to normal tissues.

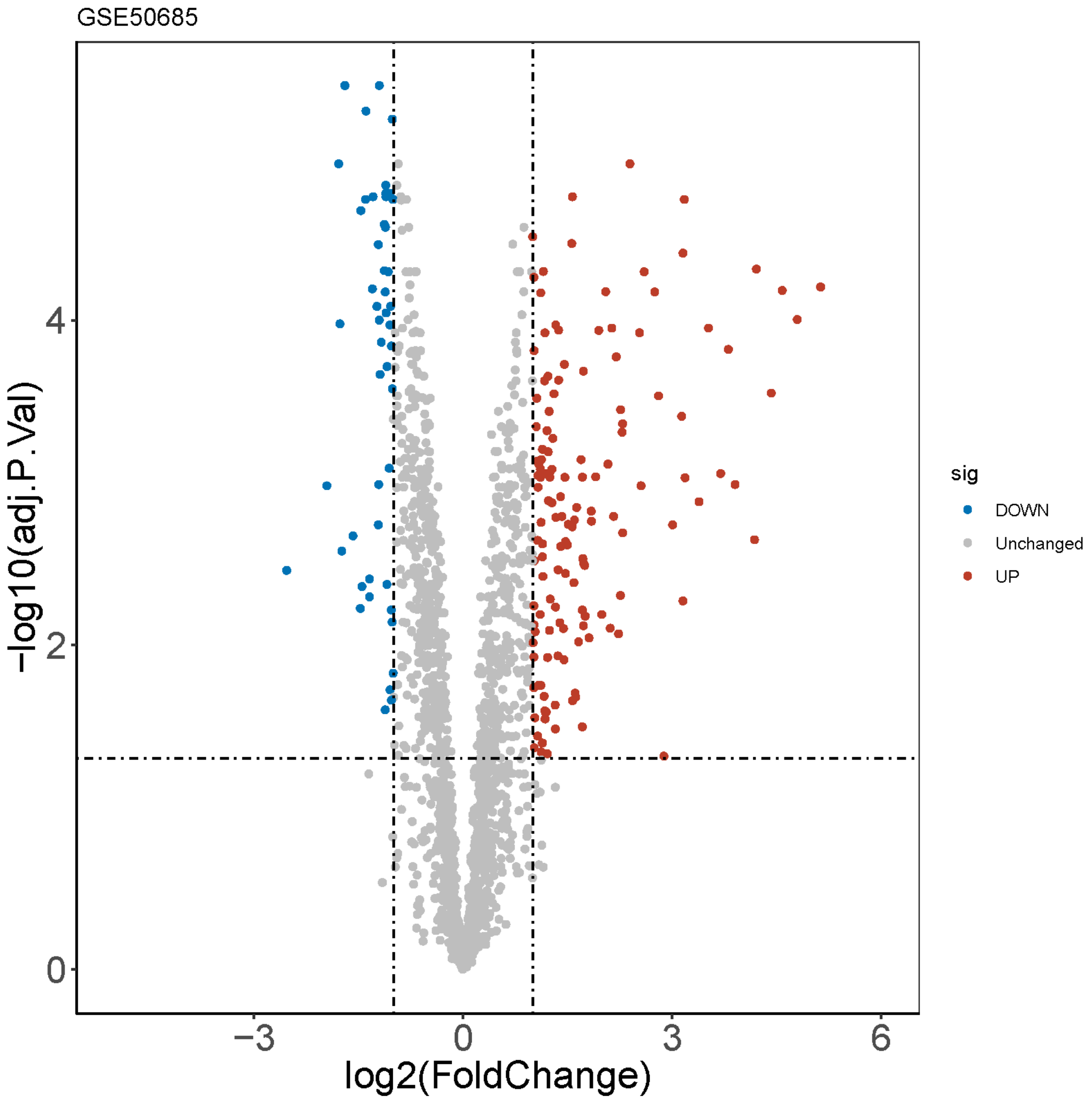

Supplementary Figure 1C. Volcano plot of the DEGs in GSE50685. The red and blue dots indicated significantly upregulated or downregulated expression of genes in mammary gland tissues with live *E. coli* infection compared to normal tissues.
